# Device modeling and numerical study of a double absorber solar cell using a variety of electron transport materials

**DOI:** 10.1016/j.heliyon.2023.e18265

**Published:** 2023-07-13

**Authors:** Sheikh Hasib Cheragee, Mohammad Jahangir Alam

**Affiliations:** Department of Electrical and Electronic Engineering, Bangladesh University of Engineering and Technology, Dhaka-1000, Bangladesh

**Keywords:** Solar cell, Double absorber, Simulation, Defect concentration, PCE, SCAPS 1D

## Abstract

In photovoltaic (PV) technology, halide perovskites are the prospective choice for highly efficient solar absorbers because of their superior optical properties, enhanced efficiency, lightweight, and low cost. In this study, a double absorber solar device using an inorganic perovskite called NaZn_0_._7_Cu_0_._3_Br_3_ as the top absorber layer and MASnI_3_ as the bottom absorber layer is analyzed utilizing the SCAPS-1D simulation tool. The primary goal of this study is to look for a device architecture with a higher efficiency level. Here, current matching over two active layers is performed by adjusting the thickness of both active layers. This research focuses on the effect of various electron transport layers, varied absorber layer thicknesses, temperatures, absorber defect density, and metalwork functions on the performance of the proposed photo-voltaic cells. After researching a variety of solar cell architectures, it is revealed that FTO/ZnO/ NaZn_0_._7_Cu_0_._3_Br_3_ / MASnI_3_ / CuO /Au arrangement has an open circuit voltage of 1.1373 V, Fill Factor of 82.13%, short circuit current density of 34.71 mA/cm^2^ and highest power conversion efficiency (PCE) of 32.42%. Here, the simulations of the device indicated that a thickness of around 1 μm for the MASnI_3_ absorber was optimum. Additionally, the results of the simulations demonstrate that the efficiency of the device rapidly drops with increasing absorbers defect density and temperature, and device structures are steady at 300 K. Finally; any conductor can make the anode if its work function is larger than or equal to 5.10 eV.

## 1 Introduction

Renewable energy sources, such as solar, are being looked at as a long-term answer for meeting the world's energy needs, and their optimal usage has the potential to reduce the harmful environmental impacts of the coal and power industries. In order to address global climate change brought on by the fossil fuel sector, high-performance, low-cost solar panel technology is now crucial [1]. However, the bandgap of the absorber material restricts the amount of light that can be absorbed, making it difficult for single-junction solar cells to reach the Shockley-Queasier limit. Firstly, Non-absorbed photons with energies below the bandgap are mostly responsible for this decline in performance. Secondly, using photon energy to produce carriers instead of free electron-hole pairs results in thermalization losses, which reduces device efficiency without increasing output. Researchers are concentrating on double-absorber solar cells to get around the Shockley-Queasier constraint. Now, it is possible to raise the efficiency of these devices, even if single-layer perovskite cells may be more effective in some cases. In this regard, Zhang et al. developed a double-absorber solar cell based on CsPbI_x_Br_3-x_/FAPbI_y_Br_3-y_ with a PCE of 17.48% [2]. However, Alzoubi et al. successfully researched double-absorber solar cells and reported that their efficiency could be increased to 19.40% [3]. Rahman et al. developed a numerical design of a double absorber solar cell based on CdTe/FeSi_2_, and achieved a power conversion efficiency of 27.35% [4]. Using SCAPS-1D, Abedini-Ahangarkola et al. researched high-efficiency perovskite/perovskite double absorber solar cells and their overall efficiency of 30.29% [5].

There has been recent interest in organometallic lead halide perovskite solar cells (PSC) as a silicon-free alternative for generating solar power and a complementary technology for silicon photovoltaics [6,7]. Despite their superior performance, lead-based perovskites are hazardous and unstable [7]. However, tin halide perovskite is one of the most promising choices for creating lead-free PSC [8]. Compared to lead-based analogs, higher theoretical PCE, higher carrier mobilities, and better light absorption are all possible in tin halide-based perovskites [9]. Perovskites based on NaZnBr_3_ have been developed to solve the problems of instability and inefficiency; one such material is NaZn_0_._7_Cu_0_._3_Br_3_ [10].

Perovskite solar cells are stable and repeatable due to their chemical inertness, effective hole-blocking at the interface, efficient charge transport, and eco-friendliness [11]. However, selecting the proper electron transport layer (ETL) and hole transport layer (HTL) of these devices can enhance performance, facilitate both electron and hole extraction from the perovskite, and make it simpler to transport through structures. Here, ZnO is utilized as ETL because of its ease of construction, low influence on hysteresis, and its excellent quality of device performance [12]. CuO was selected as the HTL because of its better charge accumulation properties, excellent hole mobility, wide accessibility band alignments that match MASnI_3_, ability to be handled in a solution, and chemically stable [13].

The SCAPS-1D modeling tool designed and simulated a novel double absorber solar cell that utilized the features of multiple perovskites by adopting a double absorber layer made of NaZn_0.7_Cu_0.3_Br_3_ and MASnI_3_ as the top and bottom absorbers, respectively. Due to the constraints of SCAPS-1D, it is assumed that there is an optically and electrically loss-free tunneling connection between the top and bottom absorbers. Several electron transport materials, including ZnO, PCBM, WO_3_, C_60_, and CdS, are utilized to determine the total effectiveness of a double absorber solar cell. The impact of operating temperature, active layer defect density, and absorber layer thicknesses on the suggested double active layer solar cell's performance is also studied. Also, the efficacy of several solar cell architectures with numerous back metal contacts (such as Cu, Zn, Fe, C, W, Au, Pd, Re, and Pt) is examined. The best double absorber structure has been found to have a PCE of 32.42%, with band gap values of 1.76 eV and 1.3 eV, respectively. This model and results will provide future experimenters with a wealth of data.

## 2 Simulation methodology and device structure

### 2.1 Simulation methodology

**Fig. 1:**
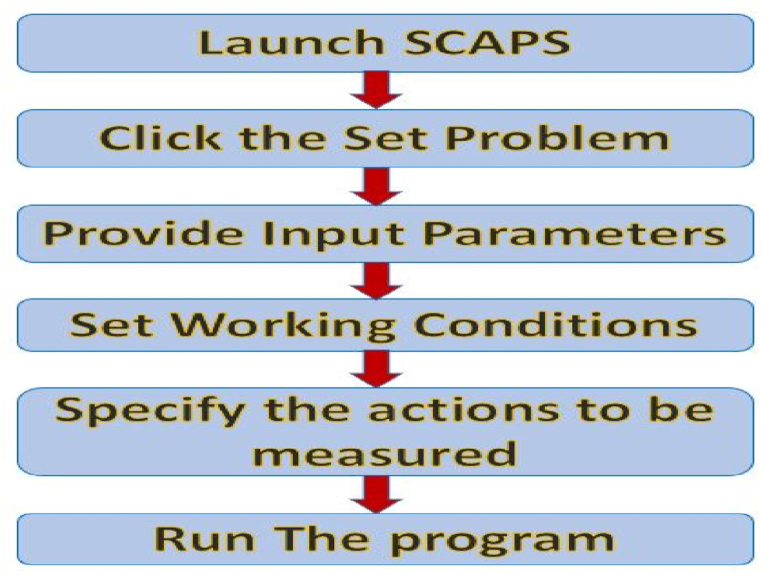
Flowchart of SCAPS-1D simulation procedure.

In this study, SCAPS-1D, a computer simulation tool has been used having version 3.3.10, that was created by Professor Marc Burgelman [14]. SCAPS-1D was chosen as our solar device simulator due to its advantages over competing programs and consistent agreement with previous research findings [15,16]. The flowchart of the SCAPS-1D simulation procedure is shown in Fig. 1. To understand the physics behind SCAPS, it is necessary to solve 1-D general semiconductor equations using the well-known transport equation, continuity equation, and Poisson's equation while additionally taking into consideration one or more recombination processes. In this case, the transport equation defines how charge carriers (electrons and holes) travel through a solar cell, and the continuity equation links the processes of carrier creation, recombination, and transport. Poisson's equation, which considers the distribution of charges and the electric field, explains the electrostatic potential distribution within a solar cell. The term “diffusivity” describes the capacity of charge carriers to disperse or spread out inside a material, while the term “diffusion length” measures the length over which diffusion is important and impacts carrier movement in a solar cell. The average amount of time that a charge carrier (electron or hole) spends recombining or disappearing in a material is known as the carrier lifetime in the context of solar cells. When there is no external load attached, the voltage between a solar cell's terminals is known as open circuit voltage. When the structure is defined by solving equations (1), (2), (3), (4), (5), (6), (7), SCAPS may be able to compute several PV performance measurements:

(1) $$Poisson\;Equations:\frac{\partial^{2}\varphi }{\partial^{2}x}=-\frac{\partial E}{\partial x}=-\frac{\rho }{\varepsilon_{s}}=-\frac{q}{\varepsilon_{s}}\left[p-n+N_{D}\left(x\right)-N_{A}\left(x\right)\pm N_{\mathrm{d}ef\left(x\right)}\right]$$

(2) $$ContinuityEquation:\frac{\partial_{n,p}}{\partial t}=\frac{1}{q}\frac{\partial J_{n}}{\partial x}+\left(G_{n}-R_{n}\right)+\frac{1}{q}\frac{\partial J_{p}}{\partial x}+\left(G_{p}-R_{p}\right)$$

(3) $$Transport\;Equation:J_{n,p}=nq\mu_{n}E+qDn\frac{\partial n}{\partial x}+pq\mu_{p}E+qDp\frac{\partial p}{\partial \varkappa }$$

(4) $$Diffusivity:D_{n,p}=\left[\left(\frac{k_{B}T}{q}\right)\mu_{n,p}\right]$$

(5) $$Diffusion\;length:L_{n,p}=\sqrt{D_{n,p}\tau_{n,p}}$$

(6) $$Open\;circuit\;voltage:v_{oc}=\frac{nk_{B}T}{q}\left[\ln\left(\frac{I_{L}}{I_{0}}+1\right)\right]$$

(7) $$Carrier\;lifetime:\tau =\frac{1}{\sigma N_{t}v_{t\dot{h}}}$$

Where φ = cells electrostatic potential, ε = material's permittivity, E stands for the electric field, n & p = concentrations of free electrons & holes, $N_{A}$ & $N_{D}$ = concentrations of doping, N_def_ is the concentration of probable defects, *ρ* and q are the elemental charges, $\mu_{n,p}$ stands for “mobility of electron or hole, $G_{n,p}$ = rate of the optical generation, $\tau_{n,p}$ stands for “lifetime of electron or hole, $\frac{\partial_{n,p}}{\partial x}$ stands for “concentration gradient of electron or hole, $R_{n,p}$ stands for the rate of recombination, $I_{0}$ = saturation current, $\frac{k_{B}T}{q}$ stands for thermal voltage, and $I_{L}$ = light-generated current [17].

### 2.2 Device structure

**Fig. 2:**
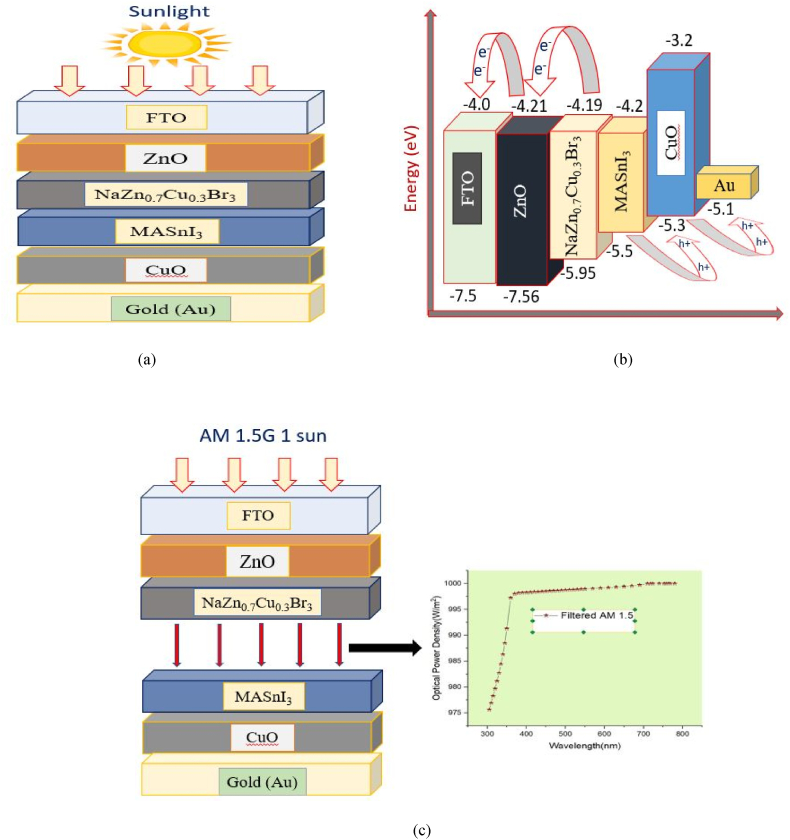
Schematic of the proposed: (a) Device structure, (b) Energy band alignment diagram (c) Device structure representation with the top section of the device lighted with AM1.5 spectrum and the bottom part illuminated by the device's filtered spectrum.

The proposed double absorbers solar cell has the following layers: FTO /ZnO / NaZn_0_._7_Cu_0_._3_Br_3_ / MASnI_3_ / CuO /Au, with FTO serving as transparent conducting oxide (TCO), ZnO serving as the ETL, NaZn_0_._7_Cu_0_._3_Br_3_ & MASnI_3_ serving as the active layers, CuO serving as the HTL, and Gold (Au) serving as the back electrode shown in Fig. 2 (a). In this case, all the simulations have been performed with the following parameters: 300K temperature, 1.0 × 10^6^ Hz frequency, and the standard illumination of AM 1.5 G 1 sun. The absorber layer thickness was optimized using simulation, while the other layers were optimized using data from the literature. In this scenario, two absorbers are linked by an ideal tunnel without electrical resistance or optical loss. To reduce shunting and shield the top layers from solvents and sputtering damage, double-absorber solar cells have a layer of conformal recombination between the layers of absorbers [5]. Fig. 2(b) illustrates the different energy levels in each layer of the proposed structure. Fig. 2(c) represents the device structure with the top section lighted with AM1.5 spectrum and the bottom part illuminated by the device's filtered spectrum. Table 1 contains a list of the electrical parameters that were utilized in the simulations, and their values were obtained from the relevant computational and experimental studies that have been published in the literature, where W is the layer thickness, ε_r_ = relative dielectric constant, *χ* = electron affinity, μ_n_ & μ_p_ = electron and hole mobility, E_g_ = bandgap, v_te_, v_tp_ are the thermal velocity, N_D_ & N_A_ = donor and acceptor densities, N_v_ & N_c_ = the valence band and conduction band's respective effective state densities correspondingly, and N_t_ stands for total defect concentration. Moreover, Table 2 displays the interface characteristics of the ETL/ NaZn_0_._7_Cu_0_._3_Br_3_ and MASnI_3_ /HTL systems. This enables us to examine the effect of defect concentration while maintaining a constant level of overall defect density at the interface.

**Table 1:** The following is a list of the parameters that were used to run this simulation.

Parameters	FTO [18]	ZnO [12]	NaZn_0.7_Cu_0.3_Br_3_ [10]	MASnI_3_ [19]	CuO [20]
W (μm)	0.5	0.05	0.02	1.0	0.30
E_g_ (eV)	3.50	3.35	1.76	1.3	2.1
χ (eV)	4.00	4.21	4.19	4.2	3.2
Є_r_	9.00	9.00	5.80	8.2	7.11
N_c_ (1/cm^3^)	2.20 × 10^18^	2.20 × 10^18^	1 × 10^16^	1 × 10^18^	2.2 × 10^18^
N_v_ (1/cm^3^)	1.80 × 10^19^	1.80 × 10^19^	1 × 10^16^	1 × 10^18^	1.80 × 10^18^
V_te_ (cms^−1^)	1E+7	1 × 10^7^	1 × 10^7^	1 × 10^7^	1E+7
V_tp_ (cms^−1^)	1 × 10^7^	1 × 10^7^	1E+7	1 × 10^7^	1E+7
μ_n_ (cm^2^/Vs)	2E+1	2.5E+1	20	1.6	3.4
μ_p_(cm^2^/Vs)	1E+1	1E+2	20	1.6	3.4
N_D_ (1/cm^3^)	2E+19	1E+18	1 × 10^19^	0	0
N_A_ (1/cm^3^)	0	0	1 × 10^19^	1 × 10^14^	1 × 10^20^
N_t_ (1/cm^3^)	1E+15	1 × 10^14^	1 × 10^14^	1 × 10^13^	1E+15

**Table 2:** The parameters for the interface defects used in this simulation.

Parameters	ETM / NaZn_0.7_Cu_0.3_Br_3_	MASnI_3_/ HTM
Defect Type	Neutral	Neutral
Ae (cm^2^) (Cross Section area of electrons)	1.0 × 10^−19^	1.0 × 10^−19^
Ah (cm^2^) (Cross Section area of holes)	1.0 × 10^−19^	1.0 × 10^−19^
Energetic Distribution	Single	Single
Reference for defect energy level E_t_	Above the highest E_v_	Above the highest E_v_
Energy with respect to a reference (eV)	0.600	0.600
Total density (cm^−3^)	1 × 10^10^	1 × 10^10^

## 3 Result and discussions

### 3.1 Perovskite solar cells (PSCs) with double and single active layers and their effects

**Fig. 3:**
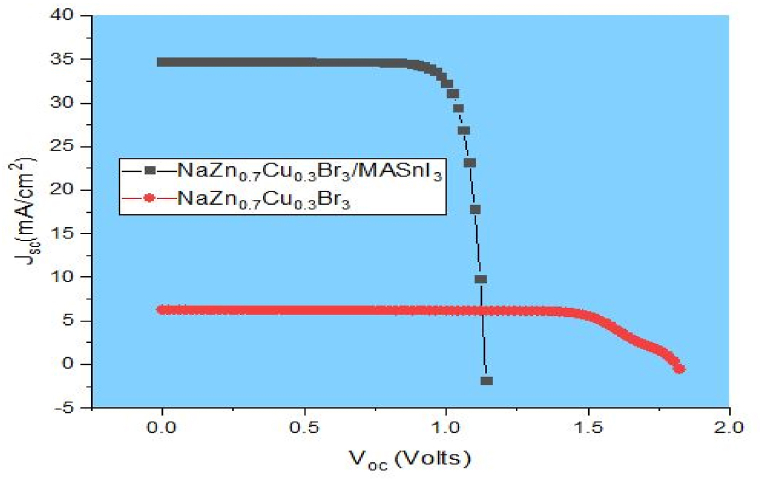
J-V curve for perovskite solar cells with a double and single absorber.

This study investigates the effectiveness of perovskite solar cells equipped with the two absorber layers depicted in Fig. 2(a). To further illustrate the performance enhancement of the proposed double absorber arrangement, it also simulated PSCs with a single (NaZn_0.7_Cu_0.3_Br_3_) active layer. Table 3 shows the outcomes of our simulations for each cell's performance characteristics. According to Table 3, the open circuit voltage of a double absorber (NaZn_0_._7_Cu_0.3_Br_3_ / MASnI_3_) PSC is reduced to 1.1373 V from 1.8098 V for a single absorber PSC. This slight decrease in Voc might be attributable to the lower bandgap of the MASnI_3_, which affects the internal potential. Moreover, the double-active layer PSCs have a significantly higher J_sc_ than their single-layer counterparts. This is because of the combinational absorber layer, which boosts photogeneration throughout a broader range of the sun's rays. Moreover, as the number of active layers expands, the fill factor for shunt routes also grows, from 75.37% for single-absorber PSC structures to 82.13% for double-absorber designs. Due to its enhanced J_sc_, Table 3 shows that our proposed double active layer device has a higher PCE percentage (32.42%). Current density versus voltage characteristics for single and double absorber layers PSCs are shown in Fig. 3. According to a comparison of J-V curves; the double-layer structure performs better because of its greater J_sc_ and somewhat lower voltages than the single-layer device. As demonstrated in Table 3, the greater photogeneration in the double absorber cells is consistent with the improved J_sc_ of such structures over their single absorber equivalent.

**Table 3:** Output metrics for PSC architectures with single and double absorber layers.

Configuration	J_sc_ (mAcm^−2^)	V_oc_ (V)	FF (%)	PCE (%)
FTO/ ZnO/ NaZn_0.7_Cu_0.3_Br_3_ /CuO/ Au	6.2751	1.8098	75.37	8.56
FTO/ ZnO/ NaZn_0.7_Cu_0.3_Br_3_ / MASnI_3_/CuO/ Au	34.7132	1.1373	82.13	32.42

**Fig. 4:**
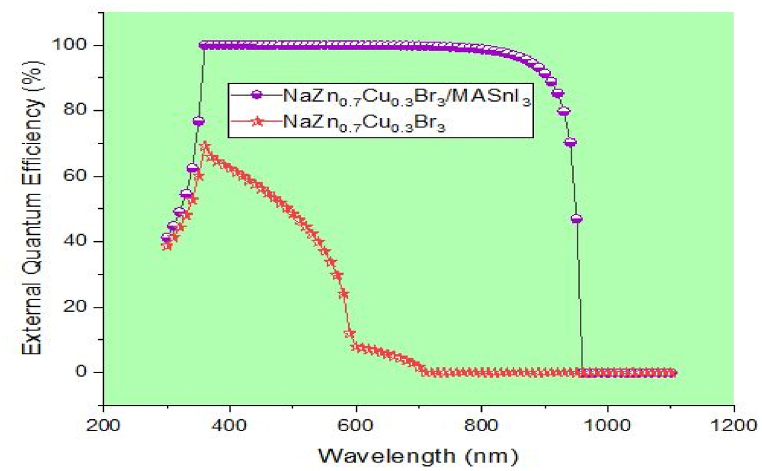
Quantum efficiency spectra for single and double absorber structures.

Fig. 4 illustrates the relationship between the solar cells' external quantum efficiency (EQE) and the wavelengths ranging from 300 to 1100 nm. NaZn_0.7_Cu_0.3_Br_3_ has a substantial bandgap (1.76 eV); hence at 710 nm, the EQE of a single absorber cell is predicted to be close to zero. In the case of a double absorber solar cell, the cut-off wavelengths are shifted to around 960 nm due to the adoption of a narrower bandgap (MASnI_3_, with an E_g_ value of 1.30 eV), which widens the EQE spectra. The effectiveness of the proposed PSC is evaluated in the following part of this article, with special attention paid to the double absorber PSC, which is the most effective construction. These variables include carrier transport materials, active layer defect density, back contact work function, working temperature, and active layer thickness.

### 3.2 The influence of a variety of ETL on proposed double absorber solar cell

**Fig. 5:**
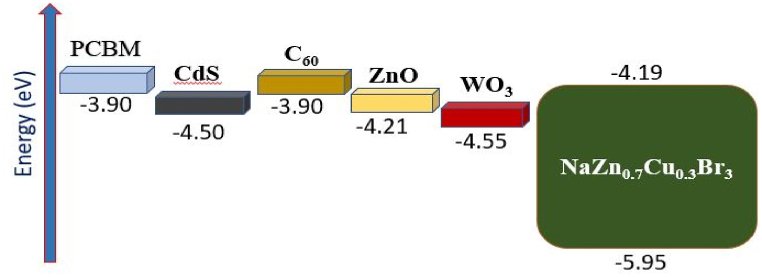
Band alignment of the NaZn_0_._7_Cu_0_._3_Br_3_ absorber with various electron transport layers.

**Fig. 6:**
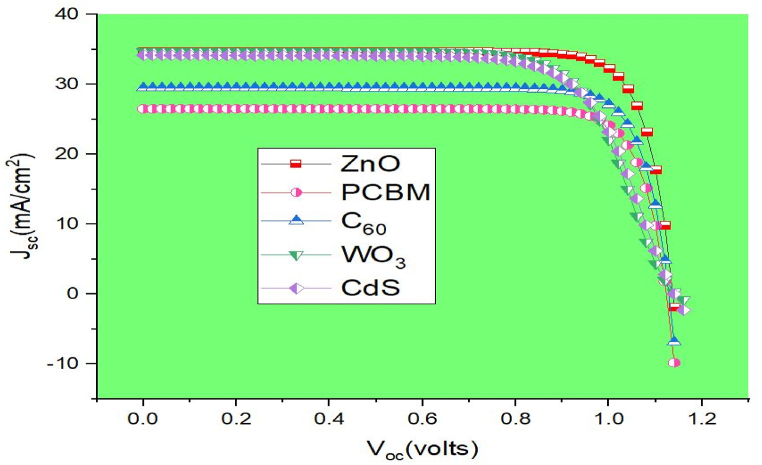
J-V plot of solar cell for several ETL materials in a double-absorber configuration.

Here, several different ETLs are investigated using CuO as the HTL. As ETL is crucial for ensuring efficient electron extraction from the absorber to the front contact, selecting an appropriate ETL material may significantly boost efficiency. Furthermore, the HTL layer is vital because it facilitates efficient hole extraction from the absorber material in the direction of the back contact. Band diagrams for several ETLs with NaZn_0_._7_Cu_0_._3_Br_3_ as the top absorber are shown in Fig. 5. Table 4 provides a summary of simulation parameters taken from the literature for a variety of ETL materials, including ZnO, PCBM, WO_3_, C_60_, and CdS, to visualize the effect of these materials on device performance. Table 5 shows that ZnO has the highest efficiency (32.42%) among the several ETLs employed in double absorber structures because of its strong band alignment, long carrier lifetime, and reduced positive conduction band offset (CBO) values with the NaZn_0_._7_Cu_0_._3_Br_3_ absorber layer. Besides, proposed arrangement shows the best output performance because of choosing proper HTL materals i. e, CuO, which has better charge accumulation properties, greater hole mobility, and proper band alignments with the MASnI_3_ absorber. Additionally, appropriate back contact material i. e, Gold (Au) also leads device efficiency greater with ensuring efficient built-in -voltage. The J-V curve of a double absorber solar cell using several ETL layers is shown in Fig. 6.

**Table 4:** The numerical parameters that were considered for each ETL material.

Parameters	ZnO [12]	PCBM [5]	C_60_ [21]	WO_3_ [22]	CdS [23]
W (μm)	0.05	0.2	0.05	0.10	0.10
E_g_ (eV)	3.35	2.0	1.7	3.15	2.4
χ (eV)	4.21	3.90	3.9	4.55	4.5
Є_r_	9.00	3.90	4.2	10.0	10.0
N_c_ (1/cm^3^)	2.20 × 10^18^	2.5 × 10^21^	8 × 10^19^	4.2 × 10^18^	2.2 × 10^18^
N_v_ (1/cm^3^)	1.80 × 10^19^	2.5 × 10^21^	8 × 10^19^	9 × 10^18^	1.8 × 10^19^
V_te_ (cms^−1^)	1 × 10^7^	1 × 10^7^	1E+7	1 × 10^7^	1E+7
V_tp_ (cms^−1^)	1 × 10^7^	1 × 10^7^	1E+7	1 × 10^7^	1E+7
μ_n_ (cm^2^/Vs)	2.5E+1	2 × 10^−2^	8 × 10^−2^	20	350
μ_p_ (cm^2^/Vs)	1E+2	2 × 10^−2^	3.5 × 10^−3^	10	25
N_D_ (cm^−3^)	1E+18	1 × 10^19^	2.6 × 10^18^	2 × 10^18^	1 × 10^17^
N_A_ (cm^−3^)	0	0	0	0	0
N_t_ (cm^−3^)	1 × 10^14^	1 × 10^15^	1 × 10^14^	1 × 10^14^	1 × 10^14^

**Table 5:** Comparing the different ETL's impact on the proposed double absorber device topologies.

Double absorber configuration	J_sc_ (mAcm^−2^)	V_oc_ (V)	FF (%)	PCE (%)
FTO/ ZnO/ NaZn_0.7_Cu_0.3_Br_3_ / MASnI_3_/CuO/ Au	34.7132	1.1373	82.13	32.42
FTO/ PCBM/ NaZn_0.7_Cu_0.3_Br_3_ / MASnI_3_/CuO/ Au	26.5006	1.1236	82.02	24.42
FTO/ C_60_/ NaZn_0.7_Cu_0.3_Br_3_ / MASnI_3_/CuO/ Au	29.4746	1.1290	82.13	27.33
FTO/ WO_3_ / NaZn_0.7_Cu_0.3_Br_3_ / MASnI_3_/CuO/ Au	34.7174	1.1443	71.59	28.44
FTO/ CdS / NaZn_0.7_Cu_0.3_Br_3_ / MASnI_3_/CuO/ Au	34.1668	1.1402	71.44	27.23

### 3.3 The impact of absorber thickness on photovoltaic output in double absorber solar cells

**Fig. 7:**
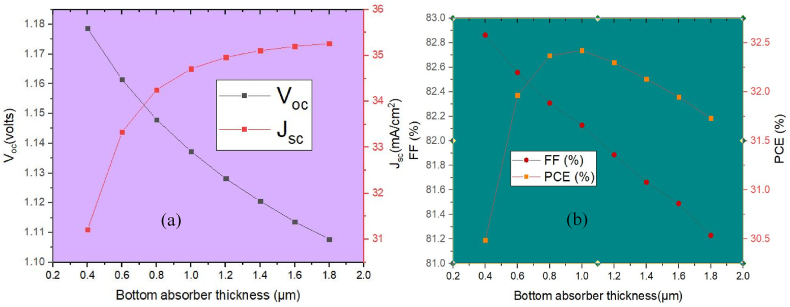
Effect of absorber thickness on (a) current-voltage curve, (b) FF, and PCE.

The absorber material and changes in its thickness are crucial to a photovoltaic cell's efficiency. Therefore, the thickness of the solar cell needs to be greater than the diffusion lengths of such charge-generated particles in order to achieve successful harvesting. Absorption and recombination should be able to equalise the photo-generated holes and electrons [24]. Usually, the absorber layer thickness facilitates charge carrier transmission to ETLs and HTLs. Consequently, increasing the thickness improves the device's effectiveness by absorbing more photon light [25]. However, as thickness was increased, recombination along the ETL/ NaZn_0.7_Cu_0.3_Br_3_ and MASnI_3_ /HTL interfaces elevated, leading to decreased photovoltaic performance [26]. The effectiveness of a solar cell having double absorber was evaluated by changing the bottom absorber thickness ranging from 0.4 μm to 1.8 μm while keeping the thickness of all other layers constants. The correlation between absorber thickness, V_oc_, and J_sc_ is depicted in Fig. 7(a). Here, Fig. 7(a) shows that the value of J_sc_ is 31.21 mA/cm^2^ and V_oc_ is 1.18 V at a thickness of 0.4 μm, and then V_oc_ values decline to 1.11 V, and J_sc_ raised to 35.26 mA/cm^2^ with a thickness of 1.8 μm. This means carrier recombination rises with increasing absorber thickness, leading to a drastic drop in V_oc_. Additionally, J_sc_ increases as the absorber thickness grows due to higher charge density and light absorption, which drives up J_sc_ due to the strong absorption coefficient. Fig. 7(b) depicts the FF and PCE curves for a range of absorber thicknesses, showing that FF rises with increasing thickness due to increased resistance. Fig. 7(b) also demonstrates that PCE grows up to its maximum at a thickness of 1.0 μm, after which it drops off significantly. According to studies, the most efficient thickness for the bottom absorber layer is 1 μm to achieve greater efficiency.

### 3.4 The influence of bulk defect concentration on the effectiveness of the proposed double-absorber solar cells

The study of defects is essential for the device's performance. When more defects in the absorber material per unit area, more pinholes develop, the film deteriorates faster, and the device's stability and effectiveness suffer [27]. Defects are categorized as shallow or deep based on their position and depth. The shallow defects density is in the order of 1E10-1E13 cm^3^; however, the deep defects density is in the order of 1E14 cm^3^ and can reach up to 1E16 cm^3^ in size [28]. When the absorber layer defect density is greater, the SRH model predicts that the quality of the layered materials will decline, carriers will generate recombinants, and the lifetime will be cut short. Using Eqns. (8), (9), we examined the effect of defect concentrations in different layers on the overall structure [29].

(8) $$\tau_{n,p}=\frac{1}{\sigma v_{th,n,p}Nt}$$

(9) $$R_{SRH}=\frac{np-n_{i}^{2}}{\tau_{p}\left(n+n_{i}\right)+\tau_{n}\left(p+p_{i}\right)}$$

Where,

$R_{SRH}$ is the Shockley Read Hall recombination rate, n and p = electron and hole concentration, $\tau_{p}$, $\tau_{n}$ = lifetime of electron and hole, N_t_ = Total defect concentration, V_th_ = Thermal velocity and n_i_, p_i_ = electron and hole's intrinsic concentration.

**Fig. 8:**
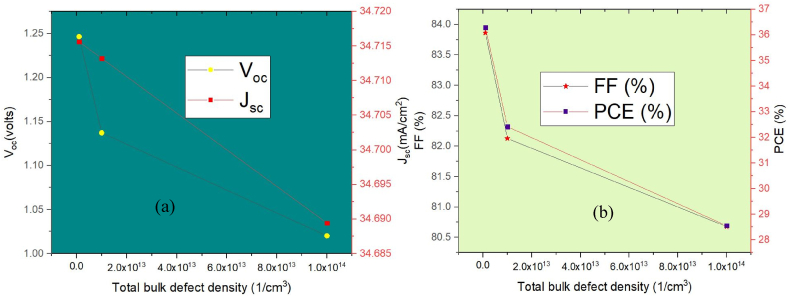
Effect of bottom absorber layer bulk defect on (a) J-V Curve (b) Fill Factor & PCE.

To investigate the impact of defects on device effectiveness, it is changed the defect concentration at the bottom absorber material ranged from 1 × 10^12^ cm^−3^ and 1 × 10^16^ cm^−3^. In this case, the top absorber layer has a defect density of 1 × 10^14^ cm^−3,^ and changing this value had no noticeable impact on the device's effectiveness. The Cell's J_sc_, V_oc_, FF, and PCE after a change of defect concentration in MASnI_3_ are depicted in Fig. 8. Fig. 8(a) illustrates that as the defect concentration of MASnI_3_ rises, the device's efficacy declines due to more substantial SRH recombination and reduced carrier lifetime, resulting in drops in J_sc_ and V_oc_. Fig. 8 shows that J_sc_ and V_oc_ of the cell decrease from 34.71 mA/cm^2^ to 32.24 mA per square centimeter and 1.24 V to 0.813 V, respectively, while PCE and FF decrease from 36.29% to 15.66% and 83.86% to 59.74% correspondingly, because of increased resistance as shown in Fig. 8(b). For bulk defect densities below 1 × 10^13^ cm^−3^, the defect concentration has no noticeable impact on the power-consumption efficiency (PCE). Based on these considerations, we determine that a bulk defect concentration of 1 × 10^13^ cm^−3^ yields the best outcomes, as indicated by the following values: V_oc_ = 1.1373 V, J_sc_ = 34.71 mA/cm^2^, FF = 82.13%, and PCE = 32.42%.

### 3.5 Temperature's impact on the performance of the proposed double-absorber solar cell

**Fig. 9:**
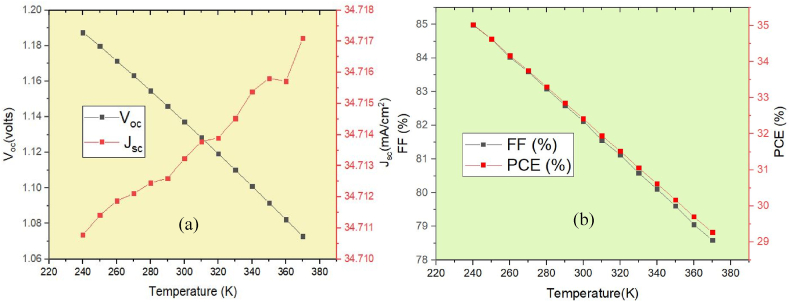
Effect of working temperature on the proposed device: (a) J_sc_ & V_oc_ (b) PCE & FF.

A temperature of 300 K (27 °C) was used as the starting point for all prior simulation investigations. Since solar cell are positioned outside, so temperature variations would impact on solar cells. Thus, it is crucial to research how temperature affects the efficiency of currently used devices. The simulation model is run at varying temperatures (from 230K to 370K) under constant light irradiation (1000 W/m^2^) to examine the influence of temperature on the effectiveness of the proposed double absorber solar cell. Fig. 9(a) and (b) depict the output device characteristics due to temperature. With increasing operating temperature, these data show a decrease in cell performance. Fig. 9(a) demonstrates that as the temperature increased, V_oc_ significantly decreased, going from 1.19 V to 1.07 V. Nonetheless, the material's band gap will reduce as the temperature rises, and increases the value of J_sc_. Fig. 9(b) shows that raising the operating temperature of the cell decreases its PCE and FF because it affects the band gap, carrier mobility, and carrier concentration, all of which are detrimental to PSC performance and efficiency. Moreover, a linear relationship exists between device performance and operating temperature, with lower operating temperatures resulting in better performance.

### 3.6 Impacts of various back contact materials on the efficiency of proposed double-absorbing solar cells

**Fig. 10:**
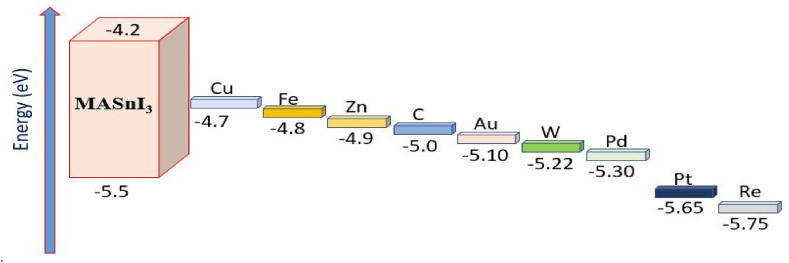
Band alignment diagram of MASnI_3_ absorber with various back contact materials.

**Fig. 11:**
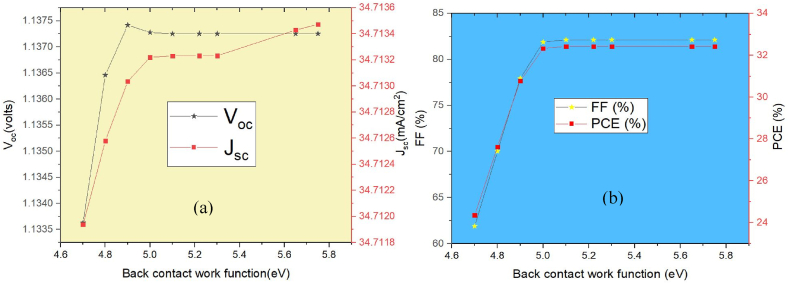
Impact of back contact materials on the performance of the proposed device: (a) V_oc_ & J_sc_ (b) FF & PCE.

The built-in voltage (V_bi_) of solar cells is greatly influenced by the back contact work function (φ), and various materials' work functions result in varied series resistances with the cell's structural elements, which influence the cell's efficiency [17]. To evaluate how the work function of metal electrodes affects photovoltaic characteristics, a variety of materials having diverse work functions are studied, including Cu (4.7 eV), Fe (4.8 eV), Zn (4.9 eV), C (5.0 eV), Au (5.1 eV), W (5.22 eV), Pd (5.3 eV), Pt (5.65 eV), and Re (5.75 eV) [17,19,[30], [31], [32]]. Energy band graphs for multiple back contacts with the absorber are shown in Fig. 10. The simulation's outcomes with the modified anode material are shown in Fig. 11. The consequence of J_sc_, and V_oc_ over diverse back contact materials is shown in Fig. 11(a), where the results in Fig. 11(b) demonstrate that the FF, and PCE significantly rise whenever the metals work function is raised ranging from 4.7 eV to 5.10 eV. This is brought on by a decreased Schottky barrier within the back contact and the hole transport material (HTM), making it easier for holes to pass from the HTM to the back contact. The performance of the cell achieves saturation at 5.10 eV. Gold has an appropriate work function, thus the reason we chose it as the back contact material in our simulation model (Fig. 1(a)). In Table 6, the PV performance of the current research is compared with that of earlier studies that have been published.

**Table 6:** PV parameters of the current work are compared to those of other double absorber structures from published literature.

Double absorber structure	J_sc_ (mA/cm^2^)	V_oc_ (V)	FF (%)		PCE(%)	References
CsPbI_x_Br_3-x_ / FAPbI_y_Br_3-y_	21.00	1.11	75.00		17.48	[2]
CZTS / Silicon (Simulation)	27.55	0.84	88.54		19.40	[3]
CdTe / FeSi_2_	49.78	0.6566	83.68		27.35	[4]
MASnI_3_ / MAPbI_3_ (Simulation)	30.87	1.15	85.29		30.29	[5]
NaZn_0.7_Cu_0.3_Br_3_ / MASnI_3_	34.98	1.0054	87.78		30.87	[33]
NaZn_0.7_Cu_0.3_Br_3_ / MASnI_3_	34.7132	1.1373	82.13		32.42	Present Work

## 4 Conclusion

In this research, a double absorber structure is numerically analyzed and optimized with the help of the SCAPS-1D simulation tools. In the first step, the effectiveness of both the double and single absorber structures is compared and studied, and the double absorber structure is shown to be more effective. Next, numerous organic-inorganic compounds are investigated as possible ETL materials for the double absorber design. The simulation findings show that ZnO works well as an ETL because of its excellent band alignment with absorbers. Additionally, the influence of the operating temperature, the absorber's thickness, back contact work function, and defect density are also investigated. Modeling has shown that metals with a work function larger than 5.10 eV are preferable for the back electrode in this double absorber configuration. It has been demonstrated that the efficiency of the device suffers a sharp decline when both the operating temperature and the absorber layer's defect concentration of the cell rise. Moreover, the defect concentration of the bottom absorber should not be greater than 1 × 10^13^ cm^3^ to attain optimal performance. The device's maximum efficiency is 32.42% at 300 K when the PCE is optimized with an absorber layer thickness of 0.02 μm for NaZn_0.7_Cu_0.3_Br_3_ and 1.0 μm for MASnI_3_, both of which are useful for experimental design in the long run.

## Author contribution statement

Sheikh Hasib Cheragee, MSc: Conceived and designed the experiments; Performed the experiments; Wrote the paper.

Mohammad Jahangir Alam, PhD: Analyzed and interpreted the data; Contributed reagents, materials, analysis tools or data.

## Data availability statement

Data will be made available on request.

## Declaration of competing interest

The authors declare that they have no known competing financial interests or personal relationships that could have appeared to influence the work reported in this paper.
